# Objective Comparison of the First-Person–View Live Streaming Method Versus Face-to-Face Teaching Method in Improving Wound Suturing Skills for Skin Closure in Surgical Clerkship Students: Randomized Controlled Trial

**DOI:** 10.2196/52631

**Published:** 2024-08-30

**Authors:** Freda Halim, Allen Widysanto, Petra Octavian Perdana Wahjoepramono, Valeska Siulinda Candrawinata, Andi Setiawan Budihardja, Andry Irawan, Taufik Sudirman, Natalia Christina, Heru Sutanto Koerniawan, Jephtah Furano Lumban Tobing, Veli Sungono, Mona Marlina, Eka Julianta Wahjoepramono

**Affiliations:** 1 Department of Surgery, Faculty of Medicine, Pelita Harapan University, Boulevard Jenderal Sudirman, Faculty of Medicine Building, 2nd Floor, Tangerang, 15811, Indonesia, 62 541-10130, 62 542-05025; 2 Department of Pulmonology and Respiratory Medicine, Faculty of Medicine, Pelita Harapan University, Tangerang, Indonesia; 3 Department of Neurosurgery, Faculty of Medicine, Pelita Harapan University, Tangerang, Indonesia; 4 Department of Oral Maxillofacial Surgery, Faculty of Medicine, Pelita Harapan University, Tangerang, Indonesia; 5 Department of Orthopaedics and Traumatology, Faculty of Medicine, Pelita Harapan University, Tangerang, Indonesia; 6 Department of Public Health, Faculty of Medicine, Pelita Harapan University, Tangerang, Indonesia; 7 Department of Medical Education, Faculty of Medicine, Pelita Harapan University, Tangerang, Indonesia

**Keywords:** teaching method, live streaming, first-person view, face-to-face, simple wound suturing

## Abstract

**Background:**

The use of digital online teaching media in improving the surgical skills of medical students is indispensable, yet it is still not widely explored objectively. The first-person–view online teaching method may be more effective as it provides more realism to surgical clerkship students in achieving basic surgical skills.

**Objective:**

This study aims to objectively assess the effectiveness of the first-person–view live streaming (LS) method using a GoPro camera compared to the standard face-to-face (FTF) teaching method in improving simple wound suturing skills in surgical clerkship students.

**Methods:**

A prospective, parallel, nonblinded, single-center, randomized controlled trial was performed. Between January and April 2023, clerkship students of the Department of Surgery, Pelita Harapan University, were randomly selected and recruited into either the LS or FTF teaching method for simple interrupted suturing skills. All the participants were assessed objectively before and 1 week after training, using the direct observational procedural skills (DOPS) method. DOPS results and poststudy questionnaires were analyzed.

**Results:**

A total of 74 students were included in this study, with 37 (50%) participants in each group. Paired analysis of each participant’s pre-experiment and postexperiment DOPS scores revealed that the LS method’s outcome is comparable to the FTF method’s outcome (LS: mean 27.5, SD 20.6 vs FTF: mean 24.4, SD 16.7; *P*=.48) in improving the students’ surgical skills.

**Conclusions:**

First-person–view LS training sessions could enhance students’ ability to master simple procedural skills such as simple wound suturing and has comparable results to the current FTF teaching method. Teaching a practical skill using the LS method also gives more confidence for the participants to perform the procedure independently. Other advantages of the LS method, such as the ability to study from outside the sterile environment, are also promising. We recommend improvements in the audiovisual quality of the camera and a stable internet connection before performing the LS teaching method.

## Introduction

Using a combination of traditional and online teaching methods in the training of medical students is unavoidable and indispensable in the 21st century, especially in the Education 4.0 framework [1]. Although blended learning methods have been applied in many disciplines, its use in surgical clerkship training has not been thoroughly explored [2,3]. This gap was made obvious during the COVID-19 pandemic, as the training of medical students in various countries was disrupted since digital online tools were not ready to be used in the medical education field [4-6].

Compounding this problem is the discrepancy between the growth rate of new medical students compared to the training rate of certified medical school lecturers [7,8]. The Indonesian Ministry of Education stated that the ideal ratio of lecturers to medical students for effective teaching is 1:5, which is not always achievable [9]. Online teaching methods are also especially useful in the operating theater environment, as the number of personnel in the operating theater must remain as few as possible to decrease the risk of surgical infections [10,11].

A proposed solution for these problems is by teaching procedural skills using live-streamed media with strict quality assurance to ensure the quality of the graduates [12,13]. In this manner, a certified lecturer could educate a number of students simultaneously, while reducing the number of people in the operating theater. While the surgeon is doing the procedure in the operating theater, the students or participants can see and learn the procedural skill in other places simultaneously via the internet [14,15]. Although a previous study by Shikino et al [16] suggested that video training of students are generally better accepted, this may not be applicable in learning a manual dexterity skill such as suturing.

The viewpoint shown in the live stream could also affect the learners’ understanding. Typically, live-streamed videos are presented in either first-person or third-person view, where a first-person view simulates the viewer being the person doing the procedure, and a third-person view shows the viewer looking at the surgeon doing the procedure from the side. In the context of surgical skills training, a first-person view could improve the students’ skills acquisition, as it provides a more realistic simulation of the procedure performed, especially concerning the hand movements, instrument handling, tissue handling, knot tying, and so on [17-19]. A first-person view could also bring the students’ viewpoint closer to the procedure compared to being there in person, as onlookers in the operating theater must maintain their distance due to hygiene and sterility issues [20].

An operator-mounted vlogging camera is also superior compared to fixed operating theater cameras, installed in the light fixtures or dedicated mounts, which require complicated installment, are not readily available in many theaters, and are less cumbersome compared to digital cameras with tripod settings [21-23]. Previous researchers have studied and published procedural learning methods using a minimalist and portable vlogging camera such as a GoPro, which could be easily brought into the operating theater, outpatient clinic, or classrooms [23-25]. This device is easily mountable and wearable, which also means that surgeons can easily wear it on their heads while operating, and a teaching assistant can help operate it with a simple click [26]. Head-mounted cameras are also easier to use and less intrusive to the operator compared to body mounts [23,27].

Previous studies have researched and published procedural learning methods using digital online platforms [6,13,28-32]. However, to our knowledge, there are still no studies that objectively evaluate the effectiveness of first-person–view live streaming (LS) methods in surgical training such as simple wound suturing, which is unique to this study. The aim of this study is to objectively assess whether performing simple wound sutures via LS using a first-person–view GoPro camera has the same effectiveness as traditional face-to-face (FTF) teaching.

Mastery in suturing skills for simple and clean wounds is a requirement for medical doctors. Simple wound suturing has internationally established techniques and assessment methods [33,34]. The most basic wound closure technique is the simple interrupted suture, which is a required skill for Indonesian medical doctors [35-37]. Objective assessment of this procedural skill is performed using the Objective Structured Clinical Examination (OSCE), which is routinely carried out at the Faculty of Medicine, Pelita Harapan University [32]. To improve participants’ skills, the direct observational procedural skills (DOPS) method has been incorporated into the curriculum [38].

## Methods

### Ethical Considerations

This study was reviewed and approved by the Pelita Harapan University Faculty of Medicine Ethical Board (ethical approval 011/K-LKJ/ETIK/I/2023). This study also has been registered at ClinicalTrials.gov (registration NCT06221917). Details about the study were explained to the participants, and informed consent were obtained from all the participants. All the data were already deidentified. No compensation was given to participants.

### Recruitment, Randomization, and Allocation

This study was a prospective, parallel, nonblinded, single-center, randomized controlled trial, conducted between January and April 2023. This study was not funded by any sponsor or institution. This study was conducted and reported in accordance with CONSORT (Consolidated Standards of Reporting Trials) guidelines [39] (Checklist 1).

A total of 74 surgical clerkship students of Pelita Harapan University were recruited as study participants based on a sample calculation from Lemeshow et al [40], from a previous study by Sakurai et al [41]. They were selected from a pool of 254 fifth- and sixth-year active clerkship students using simple computer randomization. They were in the final years of study in the Faculty of Medicine and had just begun their surgical rotation. These students had learned suturing in a clinical skills module during their second year of medical school but had no previous clinical experience of wound suturing in their clinical rotations, such as from a previous obstetrics and gynecology or surgical rotation. Participants who dropped out in the 1-week period between preintervention and postintervention time points were excluded. It was made clear to the students that their participation in this study would not affect their academic results in any way.

The students were then randomized into 2 groups: of the 74 participants, the first 37 (50%) selected by simple computer randomization were allocated to the FTF group, and the next 37 (50%) were allocated into the LS group. Each recruited participant underwent a pre-experiment simple suturing DOPS assessment with a randomly assigned clinical preceptor from the Department of Surgery. These 8 clinical preceptors are active surgical specialists and subspecialists, with previous experience in DOPS assessment and tutoring medical students. The assessment rubrics used in this study have been reviewed by the Medical Education Unit of Pelita Harapan University and were routinely used in OSCEs (Multimedia Appendix 1).

The FTF group was taught how to perform simple sutures on a mannequin, and they then watched from the side as a surgeon (FH) performed the simple suturing procedure on a real patient. FH is an assistant professor at the Faculty of Medicine and an active surgeon with more than 10 years of practice. The students were allowed to interact with the operator and ask questions.

The operator simultaneously wore a head-mounted GoPro Hero 8 device, which was performing a LS function. Two assistants, HSK and VSC, helped ensure that the audiovisual quality of the demonstration was adequate. When the visual exposure was not adequate, HSK would help by adjusting the camera [42].

The LS group was taken into a different room, and they watched the live stream from the GoPro on their own devices while being monitored by HSK or VSC. All participants were instructed to use a university Wi-Fi network to ensure connectivity. LS participants were encouraged to be actively involved in the teaching process, asking questions or giving feedback directly through a speakerphone when they were not clear regarding the demonstration or explanation.

Participants in both groups were allowed to ask the instructor to stop or redo the process. If the audiovisual quality of the live stream was poor, the camera setup was immediately modified, and the instructor would repeat the unclear teaching process to make sure every participant got the same explanation before proceeding to other steps. The live-streamed session was not recorded, and students were not allowed to record it on their device under supervision from HSK or VSC.

One week after the initial training, the participants performed a postexperiment DOPS assessment with the same examiner as the pre-experiment DOPS assessment, using the same rubric to avoid interexaminer bias. Data on the grade point average (GPA) index and frequency of self-training within a 1-week period of both groups were collected.

**Figure 1.**
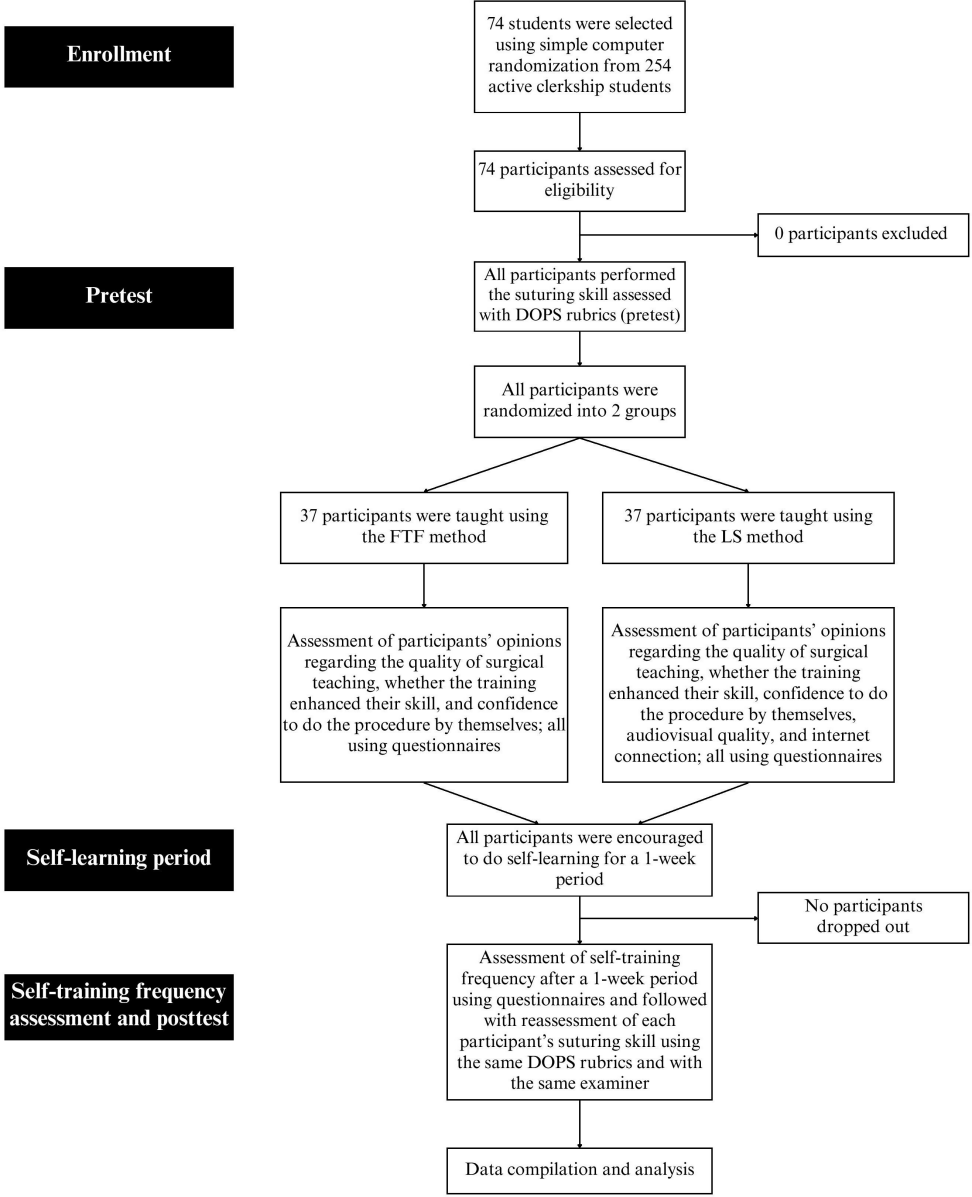
CONSORT (Consolidated Standards of Reporting Trials) diagram of participant flow. DOPS: direct observational procedural skills; FTF: face-to-face; LS: live streaming.

At the end of the teaching process, we asked both groups using a Likert-scale questionnaire for their opinion regarding the quality of surgical teaching, whether the training enhanced their skill, and the confidence of the participants to do the procedure by themselves. We also asked about the audiovisual quality of the online video as well as the internet connection for the LS group, directly after the training was finished. The participant flow is shown in Figure 1.

### Statistical Analysis

Data were analyzed using SPSS (version 23.0; IBM Corp). Paired-samples 1-tailed *t* test was used to determine the difference between the preintervention and postintervention DOPS scores. Fisher exact analysis was used to analyze the subjective evaluation of FTF versus LS effectiveness to enhance participants’ skills. Descriptive statistics were used to describe the audiovisual quality and internet connection quality.

The difference between DOPS scores (∆) was defined as the numerical difference between the scores before and after the teaching process. This numerical difference was calculated from each participant’s preintervention and postintervention scores (paired analysis). By calculating this ∆, we could objectively review the ability of the LS method compared with the traditional FTF method in enhancing suturing skills in this study.

## Results

A total of 74 study participants were included in this study, with 37 (50%) participants each in the FTF and LS groups. The characteristics of the study participants are described in Table 1. The mean GPA index of the FTF and LS groups did not show significant differences (mean 3.26, SD 0.21 vs mean 3.20, SD 0.21; *P=*.20).

**Table 1.** Study participant characteristics.

Characteristics		Value (N=74)	*P* value
**Sex, n (%)**			—^a^
	Male	26 (35)	
	Female	48 (65)	
**Age (years), mean (IQR)**		22.4 (21-26)	—
**GPA^b^ index, mean (SD)**			.20
	FTF^c^	3.26 (0.21)	
	LS^d^	3.20 (0.21)	
	Overall	3.23 (0.21)	

a Not applicable.

b GPA: grade point average.

c FTF: face-to-face.

d LS: live streaming.

Table 2 shows the objective evaluation of FTF versus LS effectiveness to enhance participants’ skill. There was a significant increase between the preintervention and postintervention DOPS evaluation scores (*P*<.001), and this difference was more apparent in the FTF group. The LS group spent significantly more time performing self-training than the FTF group (*P*=.04).

Table 3 shows the subjective evaluation of teaching method effectiveness. Most students rated the FTF or LS method as good or very good (FTF: 36/37, 97% and LS: 35/37, 95%). Most students (28/35, 76%) in the FTF group thought that the training improved their skill, while most students (24/37, 65%) in the LS group did not find the training very useful.

Table 4 shows the student assessment of LS method quality. Most students found the first-person–view quality to be good or passable (30/37, 81%). Most students (36/37, 97%) had good or acceptable internet connection, while 1 (3%) student had frequent disconnections.

**Table 2.** Objective evaluation of FTF^a^ versus LS^b^ effectiveness to enhance participants’ skill.

Variable		Value, mean (SD)	Value, range	*P* value^c^
**Overall DOPS^d^ score**				*<.001*
	Preintervention	56.7 (19.5)	15-91.7	
	Postintervention	82.7 (13.9)	41.7-100	
**Preintervention score**				.33
	FTF	58.9 (21.8)	15-91.7	
	LS	54.5 (17.1)	20-91.7	
**Postintervention score**				*.02*
	FTF	86.4 (11)	58.3-100	
	LS	78.9 (15.5)	41.7-100	
**FTF group score**				*<.001*
	Preintervention	58.9 (21.8)	15-91.7	
	Postintervention	86.44 (11)	58.33-100	
**LS group score**				*<.001*
	Preintervention	54.5 (17)	20-91.7	
	Postintervention	78.9 (15.5)	41.67-100	
**Difference between preintervention and postintervention scores (∆)**				.48
	FTF	27.5 (20.6)	0-76.6	
	LS	24.4 (16.7)	16.6-63.3	
**Total self-training frequency in 1 week**				*.048*
	LS	6.3 (3.4)	2-20	
	FTF	4.9 (2.3)	0-12	

a FTF: face-to-face.

b LS: live streaming.

c Mean difference by 1-tailed *t* test.

d DOPS: direct observational procedural skills.

**Table 3.** Subjective evaluation of FTF^a^ versus LS^b^ effectiveness to enhance participants’ skill.

Variable	FTF method (n=37), n (%)		LS method (n=37), n (%)		*P* value
**Teaching quality from instructor**					*.02*
Very good	29 (78)		18 (49)		
Good	7 (19)		17 (46)		
Passable	1 (3)		0 (0)		
Poor	0 (0)		2 (5)		
**Does the training improve your skill?**					*<.001*
Yes, it improves my skill a lot	26 (70)		7 (19)		
Yes, it does	2 (5)		6 (16)		
Not too much	9 (24)		21 (57)		
No, it doesn’t improve my skill at all	0 (0)		3 (8)		
**Confidence in doing the procedure by themselves**					*<.001*
Very confident	0 (0)		2 (5)		
Confident	24 (65)		34 (92)		
Not confident	13 (35)		1 (3)		

a FTF: face-to-face.

b LS: live streaming.

**Table 4.** Subjective evaluation of audiovisual quality and internet connection quality for the live streaming group.

Variable	Value (n=37), n (%)	
**Audiovisual quality of live streaming**		
Very good	5 (14)	
Good	17 (47)	
Passable	13 (36)	
Poor	1 (3)	
**Internet connection quality**		
Good	25 (68)	
Passable (some signal disconnections)	11 (29)	
Poor (frequent signal disconnections)	1 (3)	

## Discussion

### Principal Findings

This study aims to prove that first-person–view LS teaching has the same effectiveness compared to traditional FTF teaching in enhancing medical students’ practical skills in performing simple wound suturing. As of this writing, no other study has compared these methods before.

We considered these 2 groups to have equal basic abilities prior to their training, as their GPA index and preintervention scores were similar. It is good to see that the overall DOPS scores increased significantly between the preintervention and postintervention periods (*P*<.001), suggesting that the training process generally had good results in enhancing participants’ skills regardless of their training method.

**Figure 2.**
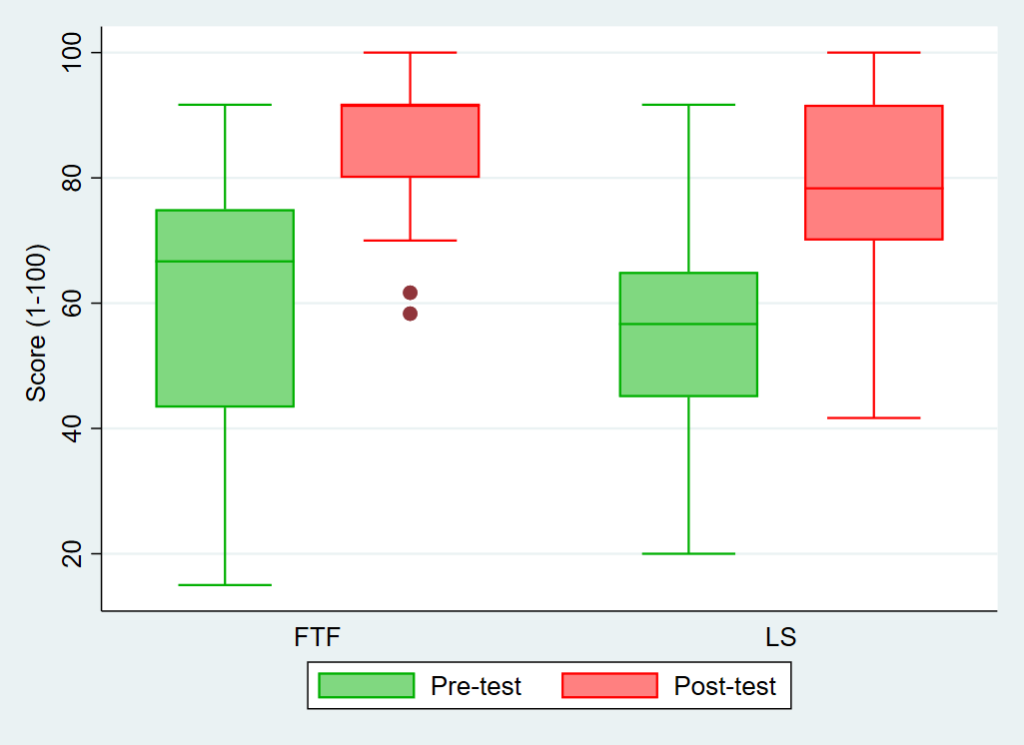
Box-plot graph of pretest and posttest scores of FTF versus LS group. FTF: face-to-face; LS: live streaming.

However, the posttest scores of the FTF participants were significantly better than those of the LS participants (FTF: mean 86.4, SD 1 vs LS: mean 78.9, SD 15.5; *P*=.02). As seen on the box-plot graph, the data variation in the LS group is wider than that in the FTF group (Figure 2, pink box plot). This wide range of data suggests significant variability in the results in the LS group, ranging from high to poor values (score).

**Figure 3.**
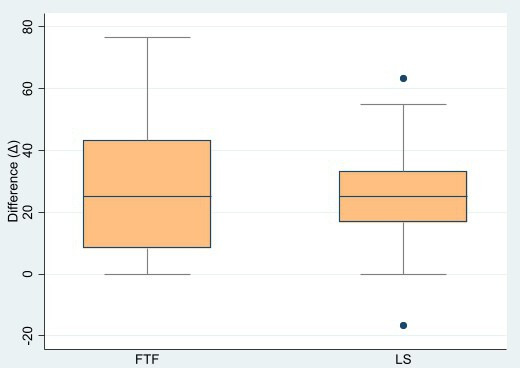
Box-plot graph of numerical differences of both groups’ scores. FTF: face-to-face; LS: live streaming.

We compared the ability of the LS method to enhance the participants’ skills with the FTF method by performing a paired analysis of the numerical differences between each participant’s preintervention and postintervention scores (∆). Based on this analysis, we found that the score increase between the FTF and LS groups was not significantly different (FTF: mean 27.5, SD 20.6 vs LS: mean 24.4, SD 16.7; *P*=.48). Nevertheless, when we observed the data variation as depicted in box-plot graph (Figure 3), we noted that the data spread of the LS group was narrower in its numerical differences compared to the FTF group, which suggested more limited ability of the LS method to enhance participants’ procedural skills compared to the FTF method. The mean score of the 2 groups were 27.5 (SD 20.6) for the FTF group and 24.4 (SD 16.7) for the LS group, which showed that the FTF group had higher score differences than the LS group. Therefore, we deduced that the LS method was still inferior to the FTF method in enhancing participants’ ability to do simple procedural skills.

Procedural skills differ from cognitive matters as they need to be mastered and self-trained within some period. We encouraged the participants to train themselves as often as possible in a 1-week period. In an effort to reduce bias, we asked the participants at the end of the 1-week period about their self-training frequency during that period. This analysis showed the LS group had more self-training frequency on average (mean 6.3, SD 3.40 vs mean 4.9, SD 2.3; *P*=.048). It is debatable whether the participants in LS group performed more self-training because they felt compelled to by the LS demonstration as mentioned by Offiah et al [23] or because of something else. It is interesting to see that even though LS participants performed more self-training than FTF participants, they did not acquire the same increase in posttest DOPS scores.

The quality of the instructions given during the FTF and LS methods was also evaluated. Participants were instructed to give feedback regarding the teaching quality, asking if the instructor gave a good, clear demonstration and explanation on the technique. We found that the majority of the FTF group thought that the teaching quality was “very good” (29/37, 78%), but the LS group was dispersed in “very good” (18/37, 49%) and “good” (17/37, 46%) responses. This result may be caused by the FTF group being physically present at the room with the instructor and, therefore, feeling more at ease to ask questions in a natural manner. Although we encouraged the participants in the LS group to actively participate in training sessions, the LS group may have had questions or comments as well but did not express them simply because they felt less engaged in the LS system. The lack of social interaction, collaborative learning, and teacher-student engagement issues are known to be barriers to online learning [43]. More specifically, the poor engagement between students and instructor in LS settings was also reported in the study of Mill et al [15]. Connectivity problems may also be an issue, as 1 participant in the LS group rated their connectivity as “poor.”

Students were also subjectively asked if their method of training improved their mastery of the skill. In the LS group, most participants (21/37, 57%) said the method did not improve their skills much, while some (3/37, 8%) said it did not improve their skills at all. This contrasted sharply with the perception of the FTF group, where most participants (26/37, 70%) said the method improved their skills a lot. These results are different from the meta-analysis performed by Mao et al [44], which found that skills proficiency improvement was not significantly different between video and conventional methods. Unfortunately, we did not specifically ask which part of the teaching method that the participants were unsatisfied with.

For the LS group, we also inquired about the audiovisual quality of the LS method. Most participants answered with “good” (17/37, 47%) and “passable” (13/37, 36%), reflecting that the quality of the teaching material needed to be enhanced. In the LS method, the participants could not move their viewpoint, head, or body position to get a better picture of what is going on compared to being present in the FTF group. The GoPro itself needed to be adjusted several times during the training due to limited visual ability, causing the participants in the LS group to not see the demonstration clearly. We also thought that the visual exposure in the LS method was still lacking, even when we used the GoPro Hero 8, which came with a 4000-pixel resolution [42,45]. This experience was also noted in LS of neurosurgery cases by Jack et al [46] using the GoPro Hero 5. The LS group also mentioned of an audio delay during the live demonstration, which could be why participants’ opinions of the quality of teaching and the training ability to improve procedural skills were varied. This audio delay is a common problem with the LS method and should be minimized in the future to enhance the effectiveness of LS in teaching procedural skills [47]. Future studies may also considered virtual reality for teaching technical skills, as it is a more immersive experience for the students [48]. Perhaps it is the quality of the teaching materials that needs to be improved to enhance the first-person–view LS method results.

Finally, we asked the participants about their confidence in performing simple wound suturing by themselves after the training. Interestingly, although the majority of both groups are confident, participants of the FTF group were less confident in performing the procedure compared with the LS group (13/37, 35% vs 1/37, 3%). We previously thought that participants of the gold standard FTF teaching method would be more confident in performing the procedure, as this method gives the participant direct visualization of the procedure and better proximity to the instructor to ask questions and, therefore, would impart more confidence to perform the procedure independently. This finding may be an effect of the first-person–view LS method, since this method puts the viewers directly in the instructor’s field of view, as if they are doing the procedure themselves. This way, the participants felt as if they have done the procedure before and are more confident in performing it independently [19,49]. Another reason may be that the LS group could learn in a more relaxed setting, as they did not have the stress and tension of trying to learn a skill from inside the high-stress environment of an operating theater and, therefore, could enhance their confidence and willingness to practice [50,51].

### Limitations

Some methods in this study could be improved. Several confounding factors could not be controlled, such as the exposure of individual students to the practice of suturing when asked to assist their preceptors in surgery during their rotation, or the enthusiasm of some students to perform self-training. As such, we limited the duration between preintervention and postntervention testing to 1 week, to reduce the effects of these factors. The retention of skills over a longer period was not explored here. We were also unable to limit contact and communication between participants from both groups during the 1-week period.

We also noted that 33% (12/37) of the LS participants had a “passable” or “poor” connection when using their own mobile devices, even though the participants were encouraged to use the university internet connection. Connectivity problems need to be more stringently monitored in the future, with all students being required to connect to university Wi-Fi.

We recommend future studies to use higher-quality recording devices to improve the quality of the teaching materials. Each participant has a different learning curve, and therefore, providing a standardized recording of the procedural skill for students would be helpful in giving them a chance to review and gain confidence before they do it independently. Using a prerecorded video to standardize the teaching material could be used, as suggested by Tackett et al [52], although using recorded media will remove the interactive quality of the live-streamed, first-person–view method. The effects of the teaching method on confidence could also be explored, to see if the first-person–view method could independently increase the participants’ confidence.

### Conclusions

Using first-person–view LS teaching of simple procedural skills such as simple wound suturing could provide many benefits for the educator, students, and teaching hospital. This method is comparable to standard FTF teaching for improving the students’ skill in performing manual tasks. Teaching a practical skill using the LS method also gives more confidence for the participants to perform the procedure independently. Further improvement to the quality of the recording device, better internet connection, and better teaching materials could improve this method in the future.

## Supplementary material

10.2196/52631 Multimedia Appendix 1 Assessment rubrics.

10.2196/52631 Checklist 1 CONSORT-eHEALTH checklist (V 1.6.1).

## Abbreviations

CONSORT: Consolidated Standards of Reporting Trials
DOPS: direct observational procedural skills
FTF: face-to-face
GPA: grade point average
LS: live streaming
OSCE: Objective Structured Clinical Examination
