# Supplementary material for: Objective Comparison of the First-Person–View Live Streaming Method Versus Face-to-Face Teaching Method in Improving Wound Suturing Skills for Skin Closure in Surgical Clerkship Students: Randomized Controlled Trial
Source: JMIR Med Educ. 2024 Aug 30;10:e52631. doi: 10.2196/52631 (PMC11423551; doi:10.2196/52631)
Supplement: Multimedia Appendix 1 [file mededu-v10-e52631-s001.pdf]

Direct Observation Procedural Skill : Wound Suturing Skills for Skin Closure

1.	Alocated Time	15 minutes
2.	Assessed Level of Competence	**Level 4:** Able to demonstrate technical ability in wound suturing skills for simple skin closure competently
3.	Competence Evaluation	1. Tools preparation<br>2. Aseptic and antiseptic (including draping*)*<br>3. Instruments handling<br>4. Suturing handling<br>5. Patient communication and education skill<br>Professionalism
4.	Anatomy System Category	Skin and subcutaneous tissue
5.	Instruction for Test Participants	**CLINICAL SCENARIO:**<br>Mrs. Martini , 28 years old, complains of wound at her left palm with the length of 3 cm caused by broken glass 30 minutes before arriving at the hospital. The patient came to ED where you are the general practitioner on duty.<br>General examination: patient is cooperative, stable, vital signs within normal limits.<br>Local examination: at the left palm, there is a wound with the length of 3cm and depth of 3mm, without active bleeding.<br><br>**Participants' Tasks :**<br>1. Communicate and prepare before minor surgical procedure<br>Do simple interrupted sutures to the patient, starting from tools preparation to wound dressing
6.	Instruction for Test Examiners	**GENERAL INSTRUCTIONS**<br>1. Observe and score (0/1/2/3) for the tasks performed by the participant as well as Global Rating score appropriately on the grading paper and computer.<br>2. Avoid interruption and/or other proceedings except for the ones instructed on examiners' instruction.<br>3. Provide relevant information/results, only when it is important.
7.		**SPECIFIC INSTRUCTIONS**<br><br>1. Examiners assess preparation<br>Tools preparation: Sterile gloves, sterile gauze, sterile drape, suturing set (needle holder, anatomic forceps, blunt forceps, suture scissors, sterile container, instrument tray), suture (non-absorbable), povidone iodine, non-sterile gloves.<br>2. Examiners assess the ability of participants to differ between sterile and non-sterile<br>3. Examiners assess the ability of participants to correctly handle the instruments<br>4. Examiners assess the ability of participants to suture (simple interrupted reef knot) rapidly and correctly<br>5. Examiners assess the professionalism of participants<br><br>**Foto** 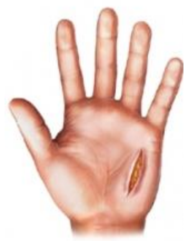
8.	Mannequin Requirement	Yes – Hecting Pad
9.	Tools Requirement	Hecting(suturing) set, Sterile gloves, sterile gauze, sterile drape with 15x15cm opening, hecting set (needle holder, anatomic forceps, chirurgic forceps, suture scissors, sterile container, instrument tray), suture (absorbable atau non absorbable), povidone iodine, non-sterile gloves, medical waste plastic bag or container
10.	Author	Freda Susana Halim, MD, Ph.D

Participant's Name :
Examiner Name:
Date of Examination :
Location:

DOPS SCORING RUBRIC

Wound Suturing Skills for Skin Closure

Scoring Rubric

COMPETENCE	0	1	2	3	WEIGHT	SCORE	Final Score (weight X Score)
1. Communication and professionalism to the patient	Participant did not ask for permission verbally and failed to do 1-2 points below thoroughly:<br>1. Carefully and thoroughly do every step as to not endanger the doctor and the patient<br>2. Pay attention to patient's comfort<br>3. Do every step based on priority<br>4. Show respect to the patient	Participant asked for permission verbally and did 1-2 points below thoroughly:<br>1. Carefully and thoroughly do every step as to not endanger the doctor and the patient<br>2. Pay attention to patient's comfort<br>3. Do every step based on priority<br>4. Show respect to the patient	Participant asked for permission verbally and did 3 points below thoroughly:<br>1. Carefully and thoroughly do every step as to not endanger the doctor and the patient<br>2. Pay attention to patient's comfort<br>3. Do every step based on priority<br>4. Show respect to the patient	Participant asked for permission verbally and did all 4 points below thoroughly:<br>1. Carefully and thoroughly do every step as to not endanger the doctor and the patient<br>2. Pay attention to patient's comfort<br>3. Do every step based on priority<br>4. Show respect to the patient	2		
2. Tools preparation	Participant did not do 6-step hand washing and/or prepare only 1-3 of the tools listed below:<br>1. Sterile surgical gloves<br>2. Needle and suture<br>3. Hecting set<br>4. Syringe<br>5. Lidocaine<br>6. Mannequin / hecting pad<br>7. Betadine<br>8. Sterile drape<br>9. Gauze and plaster	Participant did not do 6-step hand washing and/or prepare only 4-5 of the tools listed below:<br>1. Sterile surgical gloves<br>2. Needle and suture<br>3. Hecting set<br>4. Syringe<br>5. Lidocaine<br>6. Mannequin / hecting pad<br>7. Betadine<br>8. Sterile drape<br>9. Gauze and plaster	Participant did not do 6-step hand washing and/or prepare only 6-7 of the tools listed below:<br>1. Sterile surgical gloves<br>2. Needle and suture<br>3. Hecting set<br>4. Syringe<br>5. Lidocaine<br>6. Mannequin / hecting pad<br>7. Betadine<br>8. Sterile drape<br>9. Gauze and plaster	Participant did not do 6-step hand washing and/or prepare at least 8 of the tools listed below:<br>1. Sterile surgical gloves<br>2. Needle and suture<br>3. Hecting set<br>4. Syringe<br>5. Lidocaine<br>6. Mannequin / hecting pad<br>7. Betadine<br>8. Sterile drape<br>9. Gauze and plaster	3		

3. Aseptic and antiseptic (including draping)	Participant did not care for sterility	Participant did 1-2 out of 5 aseptic and antiseptic steps:<br>1. Determining sterile area and putting sterile objects inside the sterile area<br>2. Correctly wear medical gloves<br>3. Carefully maintain sterility<br>4. Sterilizing the area using betadine, in a circular motion from inward to outward, and not going back inward<br>5. Setting up sterile drape at the wound	Participant did 3-4 out of 5 aseptic and antiseptic steps:<br>1. Determining sterile area and putting sterile objects inside the sterile area<br>2. Correctly wear medical gloves<br>3. Carefully maintain sterility<br>4. Sterilizing the area using betadine, in a circular motion from inward to outward, and not going back inward<br>5. Setting up sterile drape at the wound	Participant did all of 5 aseptic and antiseptic steps:<br>1. Determining sterile area and putting sterile objects inside the sterile area<br>2. Correctly wear medical gloves<br>3. Carefully maintain sterility<br>4. Sterilizing the area using betadine, in a circular motion from inward to outward, and not going back inward<br>5. Setting up sterile drape at the wound	5		
4. Correct instruments handling	Participant did not possess any knowledge of the instruments	Participant was able to correctly handle the instruments, did 1 out of 3 listed below:<br>1. Correctly handle the forceps<br>2. Correctly handle the needle holder<br>3. Correctly picked up and handle the needle (did not handle the thread with instruments and did not handle sharps directly with hands)	Participant was able to correctly handle the instruments, did 2 out of 3 listed below:<br>1. Correctly handle the forceps<br>2. Correctly handle the needle holder<br>3. Correctly picked up and handle the needle (did not handle the thread with instruments and did not handle sharps directly with hands)	Participant was able to correctly handle the instruments, did all items listed below comprehensively:<br>1. Correctly handle the forceps<br>2. Correctly handle the needle holder<br>3. Correctly picked up and handle the needle (did not handle the thread with instruments and did not handle sharps directly with hands)	5		
5. Suturing handling and wound dressing application.	Participant did not do any of the suturing handling and technique,nor the wound dressing application.	Participant did only 2 out of 5 items listed below:<br>1. Applying local anaesthetics<br>2. Checking that the area had been anaesthetised adequately<br>3. Doing the crossing maneuver, making a reef knot<br>4. Doing simple interrupted suture **correctly** and **orderly** (**3 simple interrupted sutures** for 3cm wound)<br>5. Dressing the wound with sterile gauze and tape	Participant did only 3-4 out of 5 items listed below:<br>1. Applying local anaesthetics<br>2. Checking that the area had been anaesthetised adequately<br>3. Doing the crossing maneuver, making a reef knot<br>4. Doing simple interrupted suture **correctly** and **orderly** (**3 simple interrupted sutures** for 3cm wound)<br>5. Dressing the wound with sterile gauze and tape	Participant understood the principles of suturing and did all steps correctly starting from local anaesthetics until wound dressing (all of 5 items listed below):<br>1. Applying local anaesthetics<br>2. Checking that the area had been anaesthetised adequately<br>3. Doing the crossing maneuver, making a reef knot<br>4. Doing simple interrupted suture **correctly** and **orderly** (**3 simple interrupted sutures** for 3cm wound)<br>5. Dressing the wound with sterile gauze and tape	5		

FINAL SCORE :
Comments:

Feedback:

Sign and Name of Examiner:
